# How to predict progression-free survival in patients with grade 2 IDH-mutated diffuse gliomas after surgery: a long-term follow-up analysis

**DOI:** 10.3389/fonc.2025.1673285

**Published:** 2025-11-18

**Authors:** Marta Aprile, Vincenzo Di Nunno, Lidia Gatto, Alicia Tosoni, Damiano Balestrini, Vania Ramponi, Alfredo Conti, Monica Maffei, Stella Battaglia, Chiara Maria Argento, Enrico Zuliani, Stefania Bartolini, Marzia Margotti, Francesca Gentilini, Giovanni Tallini, Sofia Asioli, Enrico Franceschi

**Affiliations:** 1 Nervous System Medical Oncology Department, Istituto di Ricovero e Cura a Carattere Scientifico (IRCCS) Istituto delle Scienze Neurologiche di Bologna, Bellaria Hospital, Bologna, Italy; 2 Department of Radiotherapy, Azienda Unità Sanitaria Locale (AUSL)-IRCCS Scienze Neurologiche, Bologna, Italy; 3 Unit of Neurosurgery, IRCCS Istituto delle Scienze Neurologiche di Bologna, Bellaria Hospital, Bologna, Italy; 4 Department of Biomedical and Neuromotor Sciences (DIBINEM)- Alma Mater Studiorum- University of Bologna, Bologna, Italy; 5 Programma Neuroradiologia Con Tecniche Ad Elevata Complessità, IRCCS Istituto Delle Scienze Neurologiche Di Bologna Bellaria Hospital, Bologna, Italy; 6 Department of Experimental, Diagnostic and Specialty Medicine, University of Bologna, Bologna, Italy; 7 Programma Diagnostica Neuropatologica dei Tumori Cerebrali, IRCCS Istituto delle Scienze Neurologiche di Bologna, Bologna, Italy; 8 Department of Medical and Surgical Sciences (DIMEC), University of Bologna, Bologna, Italy; 9 Solid Tumor Molecular Pathology Laboratory, IRCCS Azienda Ospedaliero-Universitaria di Bologna, Bologna, Italy

**Keywords:** low grade glioma, prognostic factors, grade 2 oligodendroglioma, grade 2 astrocytoma, progression free survival

## Abstract

**Purpose:**

Available prognostic scores for adult-type diffuse glioma with isocitrate dehydrogenase (IDH) mutant were validated before the evaluation of biomolecular features. The selection of patients who did not receive postoperative radiotherapy and chemotherapy would provide an ideal setting to describe the natural history of these tumors.

**Methods:**

We investigated the clinical outcomes of patients with adult-type diffuse glioma with isocitrate dehydrogenase IDH mutation approached with active surveillance after primary surgery.

**Results:**

We evaluated 61 patients consisting of 35 patients with IDH-mutant astrocytomas and 26 patients with IDH-mutant 1p19q oligodendrogliomas. The median follow-up was 13.1 years (95% CI 11.4–17.7). A total of 56 progression-free survival events were available at the time of analysis. The median age was 32.2 years, higher in IDH-mutant 1p19q oligodendrogliomas (39.5 years) compared to IDH-mutant astrocytomas (31.4 years; p = 0.003). Residual tumor [hazard ratio (HR) 2.63, 95% CI −1.23 to 5.58, p = 0.007], post-surgical diameter product (HR 1.11, 95% CI 1–1.22, p = 0.03), and midline crossing (HR 6.79, 95% CI 1.5–30.4, p = 0.005) were the only factors directly influencing progression-free survival in univariate analyses. No variables confirmed their predictive role in multivariate models. At the time of data analysis, we registered 22 overall survival (OS) events. In a multivariate Cox regression model, histo-molecular diagnosis (oligodendroglioma *vs*. astrocytoma; HR 0.28, 95% CI 0.10–0.8, p = 0.02) and initial tumor area assessed as continuous variables (HR 1.82, 95% CI 1.01–3.3, p = 0.05) independently affected the survival of patients (p = 0.01).

**Conclusions:**

In our series, the presence and dimension of residual tumors and midline crossing were the only independent variables predicting progression-free survival after primary surgery in grade 2 diffuse glioma.

## 1 Introduction

Among primary brain tumors, adult-type diffuse glioma isocitrate dehydrogenase (IDH) mutant is a group of slow-growing, rare neoplasms (incidence of one to two cases per 100,000/year), with highly variable clinical outcomes reflecting histological and molecular heterogeneity. Over the past decade, the unequivocal impact of molecular profiling in the definition of histological tumor types has led to more accurate diagnosis and prognostic evaluation, thus raising clinical questions about appropriate management. The 2021 WHO classification of central nervous system tumors integrated IDH 1/2 mutation status as a mandatory disease-defining marker for adult diffuse gliomas (1).

IDH mutation is inversely associated with tumor grade, with the highest prevalence (~80%–85%) in adult-type diffuse glioma IDH mutant. In addition, 1p19q codeletion is a defining feature of oligodendroglioma (IDH mutant, 1p19q codeleted) compared to the astrocytoma subtype (IDH mutant, 1p19q non-codeleted) (1, 2).

In addition to clinical characteristics, both molecular features and morphological grading are associated with disease behavior. In particular, adult-type diffuse gliomas IDH mutant are characterized by indolence and favorable survival outcomes (median overall survival 10–18 years) (1–4). Nevertheless, the majority of these tumors will recur over time. A balanced consideration of the benefits expected from treatment in terms of progression-free and overall survival is necessary, considering young median age at diagnosis (30–40 years), patient long-life expectancy, and long-term side effects of adjuvant therapies. Current guidelines recommend either post-surgical surveillance or radiotherapy with a sequential PCV (procarbazine, CCNU, lomustine) regimen, calibrated on the patient's individual “risk” (5). In this context, two distinct prognostic indexes are currently being employed in clinical practice: the European Organization for Research and Treatment of Cancer (EORTC) and the Radiation Therapy Oncology Group (RTOG) criteria. Molecular typing is not incorporated within either of the two scoring systems, primarily because, at the time they were developed, the WHO classification scheme did not contemplate the integration of molecular typing with histologic diagnosis.

Whether the EORTC and RTOG risk scores can be replicated in the era of molecularly defined IDH-mutant grade 2 gliomas remains a subject of considerable debate (5–11). To date, retrospective studies have explored several prognostic indicators in adult-type diffuse glioma IDH-mutant cohorts, with inconsistent results and limitations due to heterogeneity in patient selection, diagnostic criteria adopted, and the extent of post-surgical treatment (7, 12–22). In this context, the recent results of the phase 3 practice-changing INDIGO trial provide evidence for the favorable impact of IDH1/2 inhibitor vorasidenib on progression-free survival in untreated adult-type diffuse glioma IDH-mutant patients (23). The optimal subset of patients and the appropriate timing for this new kind of treatment are matters of debate. This is because the phase 3 trial allowed randomization between 1 and 5 years after surgery. In contrast, Food and Drug Administration (FDA) approval was granted without any recommendation regarding the time from surgical treatment. Given the paradigm shift in disease profiling and the future impact of targeted therapy, it is essential to achieve a more precise definition of clinical and molecular features that affect prognosis and eventually identify tumors with different propensities to progress. With this aim, we explored prognostic factors in a cohort of adult-type diffuse glioma IDH mutant on active surveillance after primary surgery to characterize their natural history and to identify those features that could represent a rational basis for risk stratification and personalized treatment forms.

## 2 Patients and methods

### 2.1 Patients

This is a retrospective cohort study evaluating clinical and molecular variables influencing the clinical outcomes of patients with adult-type diffuse glioma IDH mutant on active surveillance after primary surgery. The primary endpoint of interest was progression-free survival (PFS), defined as the time between primary surgery and first evidence of tumor progression. The secondary endpoint of interest was overall survival (OS), defined as the time between primary surgery and death due to disease progression.

We selected patients assessed in our institution (Nervous System Medical Oncology Department, IRCCS Istituto Scienze Neurologiche di Bologna, Italy) between January 2000 and December 2021.

We selected only patients i) with a diagnosis of IDH-mutant astrocytoma (IDH-mutated/1p19q non-codeleted) and grade 2 oligodendroglioma IDH-mutated 1p19q codeleted according to the WHO 2021 classification scheme (4), ii) without evidence of contrast enhancement on the first magnetic resonance imaging (MRI), and iii) who underwent active surveillance after primary surgery. We excluded patients with known CDKN2A/B deletion, as well as those who were able to undergo oncological treatment following primary surgery, with an Eastern Cooperative Oncology Group (ECOG) score ranging from 0 to 1.

The diagnosis of diffuse astrocytoma and oligodendroglioma was confirmed after next-generation sequencing (NGS) molecular assessment and reviewed by an expert neuropathologist. Homozygous deletion of CDKN2A is defined as the simultaneous absence of signals related to the target genes in the presence of signals marking the reference chromosome in at least 30% of lesion cells.

MRIs were reviewed by an expert neuro-radiologist, who recorded the pre-surgical and post-surgical tumor/residue dimensions. MRI examination was performed, including perfusion-weighted imaging (PWI), diffusion-weighted imaging (DWI), T2-weighted fluid-attenuated inversion recovery (FLAIR) sequence, and post-contrast sequences. The extent of resection (EOR) was defined through quantitative assessment of maximal cross-sectional T2-weighted FLAIR diameters to determine the size of non-contrast-enhancing lesions. The immediate postoperative MRI scan was performed using advanced MRI techniques, within 48 hours of surgery, to evaluate EOR. Patients with gross total/complete resection were those without measurable disease, defined as lesions with clearly defined margins by MRI scan, with both perpendicular diameters on a single slice of at least 10 mm. Disease assessment was determined by the investigators according to low-grade glioma Response Assessment in Neuro-Oncology (RANO) criteria (24).

Patients were also assessed according to the prognostic risk proposed and validated by the RTOG (25) and the EORTC (26). Variables included in the RTOG and EORTC risk scores are age > 40, regardless of EOR, and age < 40 with incomplete resection for the RTOG risk score and at least three of the following: age ≥40, tumor diameter ≥6 cm, tumor crossing the midline, astrocytoma histology, and preoperative neurological deficit for the EORTC risk score.

The study was approved by the Ethical Committee of Azienda Sanitaria Locale di Bologna (protocol number CE09113, Bologna, Italy). All information regarding the human material was managed using anonymous numerical codes, and all samples were handled in compliance with the Declaration of Helsinki.

### 2.2 Molecular analysis

Molecular typing was performed on formalin-fixed and paraffin-embedded (FFPE) samples. Fluorescence in situ hybridization (FISH) was used to analyze 1p and 19q chromosomal regions. FISH was performed on 4-μm-thick sections from the most representative paraffin-embedded blocks using standard sets of 1p and 19q locus-specific identifier probes (1p36.32/1q25.2 Vysis and 19p13.2/19q13.33 Vysis) (Abbot Laboratories, Chicago, IL, USA) and following established protocols (27), as previously described (28). In cases with equivocal FISH results, 1p19q codeletion was confirmed using a single-nucleotide polymorphism (SNP) NGS panel covering the entire chromosomes 1 and 19 (28). NGS was used to identify IDH1 and IDH2 mutations. DNA for NGS was extracted after the manual dissection of tumor material under microscopic guidance from 10-μm-thick FFPE sections obtained from the same paraffin block used for FISH analysis, as previously described (28), using a laboratory-developed solid tumor multi-gene panel that includes exon 4 of both IDH1 and IDH2, where mutational hotspots are located (29). Briefly, NGS was performed using the Gene Studio S5 sequencer (Thermo Fisher Scientific), according to the manufacturer’s instructions. For amplicon library preparation, the AmpliSeq Plus LibraryKit 2.0 (Thermo Fisher Scientific) was used, starting from approximately 50 to 100 ng of input DNA. Templates were prepared using an Ion Chef Machine and sequenced using an Ion 530 chip. Sequences were analyzed with the Ion Reporter tool (Thermo Fisher Scientific). Only nucleotide variations detected in both strands and at least 5% of the total number of reads analyzed were considered for the mutational call (29). The same DNA used for NGS was also utilized to evaluate methylated–DNA–protein–cysteine methyltransferase (MGMT) promoter methylation. MGMT promoter methylation status was analyzed by pyrosequencing following established protocols (30), with a methylation cut-off at 10%. Our molecular biology laboratory employed available kits to assess the methylation status of five CpG islands located within the promoter region of exon 1 of the *MGMT* gene. The methylation percentage reported corresponds to the average methylation level across the analyzed CpG sites.

### 2.3 Statistical methods

Continuous variables were reported as mean with standard deviation, median, and range. Quantitative variables were reported as frequencies and percentages. Comparison between quantitative variables was performed using the chi-square test, while the t-test or Wilcoxon test was employed for the comparison of quantitative variables with a normal or skewed distribution.

Time to event outcomes (PFS and OS) were estimated using the Kaplan–Meier method, and the log-rank test was adopted to compare survival within different subgroups.

The impact of continuous variables on PFS and OS was further investigated using the restricted cubic spline method. Of note, as previously reported, some variables of interest, such as post-surgical residual area and initial volume area, present a skewed distribution (21, 22). To switch them to a normal distribution, base 2 logarithmic conversion was performed, as previously described (21). Multivariate analysis was performed employing a Cox regression model. The proportional hazards assumption was verified with the use of Schoenfeld residuals.

The Bayesian information criterion (BIC) and the Akaike information criterion (AIC) were used to evaluate the EORTC and RTOG predictive score (31–33). BIC and AIC are two tools that estimate predictive model performance and are based (in part) on the likelihood function. The lower the BIC and AIC detected, the better the predictive value of the model of interest. All analyses were performed using the R software version 4.3.1.

## 3 Results

### 3.1 Patient selection

Overall, we evaluated 61 patients according to inclusion/exclusion criteria (**Table 1**). This cohort consisted of 35 patients (57.4%) with IDH-mutant astrocytoma and 26 (42.6%) oligodendrogliomas IDH mutant 1p19q codeleted. The median age was 32.2 years. Patients with oligodendrogliomas IDH mutant 1p19q codeleted were significantly older (39.5 years) compared to those with IDH-mutant astrocytoma (31.4 years; p = 0.003). MGMT promoter methylation was more frequent in oligodendrogliomas IDH mutant 1p19q codeleted (80.8%) as compared to IDH-mutant astrocytomas (62.9%; p = 0.048). There were no significant differences between the distribution of the other clinical and molecular variables between the two subgroups (**Table 1**). Notably, as regards baseline disease extension, three patients with astrocytoma diagnosis had tumors crossing the midline.

**Table 1:** Clinical and molecular features of patients.

Variable	IDH-mutated 1p19q non-codeleted (N = 35)	IDH-mutated 1p19q codeleted (N = 26)	p-Value
Gender			
F	15 (42.9%)	15 (57.7%)	0.375
M	20 (57.1%)	11 (42.3%)	
Age			
Mean (SD)	31.4 (9.04)	39.5 (11.1)	0.003
Median [Min, Max]	29.7 [18.5, 63.5]	37.1 [22.7, 63.2]	
Surgery			
Partial resection	25 (71.5%)	18 (69.42%)	0.920
Complete resection	7 (20.0%)	5 (19.2%)	
Missing	3 (8.6%)	3 (11.5%)	
Pre-surgical area (mm^2^)			
Mean (SD)	1,640 (1,190)	1,890 (1,030)	0.282
Median [Min, Max]	1,180 [528, 4,620]	1,800 [180, 4,250]	
Missing	8 (22.9%)	4 (15.4%)	
Post-surgical area (mm^2^)			
Mean (SD)	263 (262)	383 (453)	0.708
Median [Min, Max]	224 [0.0, 891]	132 [0.0, 1,480]	
Missing	15 (39.5%)	12 (46.2%)	
Midline crossing			
No	30 (85.7%)	22 (84.6%)	0.396
Yes	3 (8.6%)	0 (0%)	
Missing	2 (5.7%)	4 (15.4%)	
RTOG			
High	28 (80.0%)	20 (76.9%)	1
Low	4 (11.4%)	3 (11.5%)	
Missing	3 (8.6%)	3 (11.5%)	
EORTC			
High	10 (28.6%)	3 (11.5%)	0.185
Low	16 (45.0%)	16 (61.5%)	
Missing	9 (25.7%)	7 (26.9%)	
IDH mutation			
Canonical (R132H)	32 (91.4%)	21 (80.8%)	0.403
Non-canonical (no R132H)	3 (8.6%)	5 (19.2%)	
MGMT			
Methylated	22 (62.9%)	21 (80.8%)	0.048
Unmethylated	12 (34.3%)	2 (7.7%)	
Missing	1 (2.9%)	3 (11.5%)	
Second surgery at time of tumor relapse			
Yes	22 (62.9%)	15 (57.7%)	0.563
No	9 (25.7%)	10 (38.5%)	
Missing	4 (11.4%)	1 (3.8%)	
Treatment at time of tumor relapse			
Chemotherapy	1 (2.9%)	4 (15.4%)	0.289
Radiotherapy	3 (8.6%)	3 (11.5%)	
RT → CT	3 (8.6%)	3 (11.5%)	
Surgery	5 (14.3%)	8 (30.98%)	
Surgery → CT	3 (8.6%)	1 (3.8%)	
Surgery → RT	1 (2.9%)	1 (3.8%)	
Surgery → RT → CT	14 (40.0%)	5 (19.2%)	
Missing	5 (14.3%)	1 (3.8%)	

CT, chemotherapy; EORTC, European Organisation for Research and Treatment of Cancer; IDH, isocitrate dehydrogenase; MGMT, methylated–DNA–protein–cysteine methyltransferase; RTOG, Radiation Therapy Oncology Group; RT, radiation therapy.

At the time of data analysis, the median follow-up was 13.1 years (95% CI 11.4–17.7).

### 3.2 Progression-free survival

Complete data on progression-free survival were available for 56 patients. In five patients, we were unable to obtain information about the time of progression; we thus decided to remove these patients from the PFS analyses but included them in the OS assessment. Median PFS was 3.96 years (95% CI 3.22–4.48). All patients with complete data of PFS (n = 56) experienced progression at the time of analysis.

**Figure 1:**
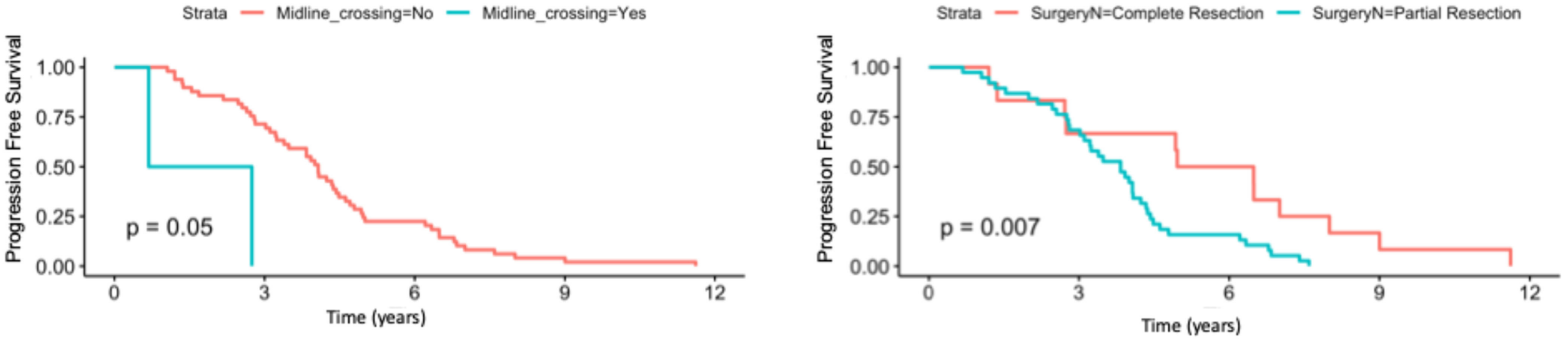
Progression-free survival according to the presence/absence of residual tumor and according to presence/absence of midline crossing.

**Figure 2:**
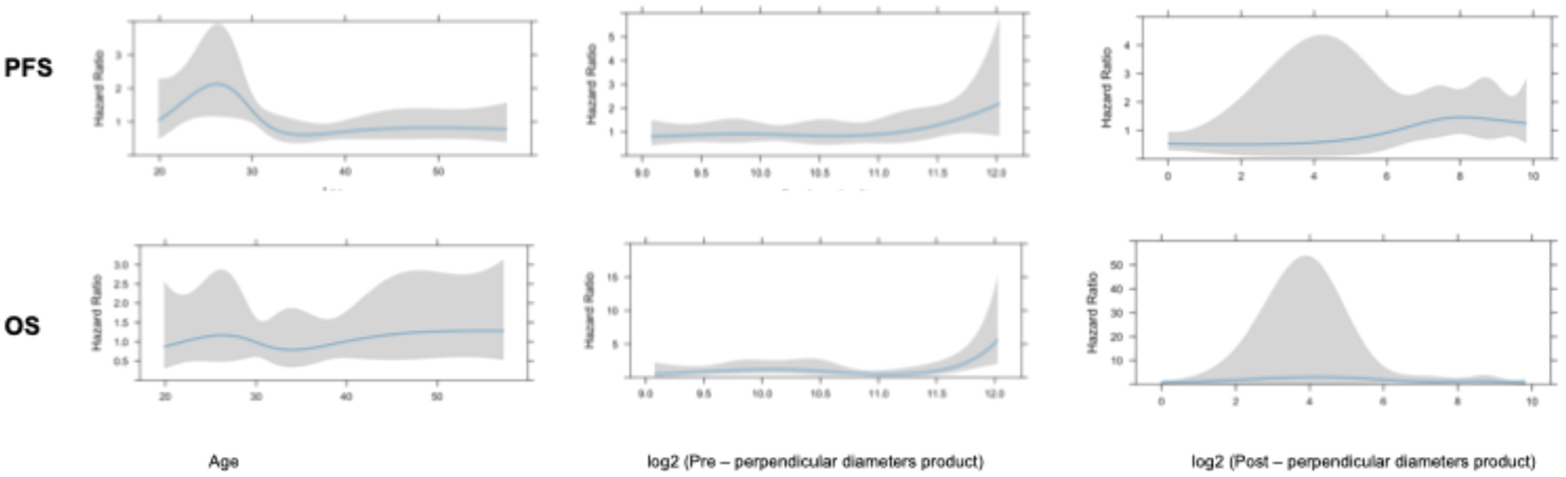
Restricted cubic splines for continuous variables.

Univariate analysis showed that midline crossing [hazard ratio (HR) 6.65, 95% CI 1.5–29.6, p = 0.004], presence of residual tumor (HR 2.63, 95% CI 1.23–5.58, p = 0.007), and post-surgical perpendicular diameter product (HR 1.11, 95% CI 1.004–1.22, p = 0.03) were the only factors affecting PFS (**Table 2**, **Figure 1**). Restricted cubic splines for continuous variables are reported in **Figure 2**. We used the restricted cubic splines in regression analysis to model non-linear relationships between a continuous predictor variable and an outcome. We investigated the impact of the following continuous variables: age (years), pre-surgical area (with log2 conversion), and residual area (with log2 conversion) on PFS. Pre-surgical area and post-surgical area larger than 2,050 and 100 mm^2^ appeared associated with a shorter PFS (**Figure 2**); however, none of these continuous variables were associated with a statistically significant impact on progression-free survival.

**Table 2:** Univariate analysis of PFS.

Variables	Overall	1p19q codeleted	1p19q non-codeleted
	Hazard ratio (95% CI) p-Value	Hazard ratio (95% CI) p-Value	Hazard ratio (95% CI) p-Value
Midline crossing (yes *vs*. no)	6.79 (1.5–30.4) p-Value = 0.05	NA	4.5 (1.1–20.68) p-Value = 0.05
Surgery (complete *vs*. other)	2.63 (1.23–5.58) p-Value = 0.007	6.51 (1.38–30.65) p-Value = 0.05	1.6 (0.66–4.14) p-Value = 0.27
Age categorical (>40 or ≤40 years)	0.72 (0.39–1.33) p-Value = 0.2	0.84 (0.36–1.95) p-Value = 0.68	0.67 (0.23–1.96) p-Value = 0.47
Histology (oligo *vs*. astro)	0.68 (0.39–1.19) p-Value = 0.2	NA	NA
RTOG (low *vs*. high risk)	0.73 (0.33–1.66) p-Value = 0.4	0.76 (0.21–2.69) p-Value = 0.67	0.7 (0.24–2.04) p-Value = 0.5
EORTC (low *vs*. high risk)	0.58 (0.29–1.15) p-Value = 0.1	0.73 (0.2–2.7) p-Value = 0.64	0.6 (0.26–1.38) p-Value =0.23
IDH mutation (canonical *vs*. non-canonical)	1.6 (0.64–3.48) p-Value = 0.3	1.56 (0.51–4.73) p-Value = 0.43	2.4 (0.7–8.39) p-Value =0.17
MGMT (methylated–unmethylated)	1.2 (0.63–2.36) p-Value = 0.7	NA	0.93 (0.44–1.95) p-Value = 0.85
Gender (male–female)	1.04 (0.61–1.7) p-Value = 0.9	1.76 (0.74–4.2) p-Value = 0.2	0.57 (0.27–1.17) p-Value = 0.12
Age continuous*	0.98 (0.95–1.008) p-Value = 0.2	0.99 (0.95–1.03) p-Value = 0.64	0.97 (0.93–1.02) p-Value = 0.26
Pre-surgical perpendicular diameter product*^	1.26 (0.87–1.80) p-Value = 0.2	1 (0.99–1.001) p-Value = 0.35	1 (0.99–1.001) p-Value = 0.1
Post-surgical perpendicular diameter product *^	1.11 (1.004–1.22) p-Value = 0.03	1.001 (0.99–1.003) p-Value = 0.07	0.99 (0.99–1.002) p-Value = 0.7

PFS, progression-free survival; RTOG, Radiation Therapy Oncology Group; EORTC, European Organisation for Research and Treatment of Cancer; IDH, isocitrate dehydrogenase; MGMT, methylated–DNA–protein–cysteine methyltransferase.

* Continuous variables

^^^ Log2 function.

When the same univariate analyses were carried out according to histology, the prognostic role of surgery was confirmed in IDH-mutant 1p19q codeleted tumors (p = 0.05) but not in 1p19q non-codeleted gliomas (0.27) (**Table 2**). The dimension of residual tumors seemed to affect the progression-free survival of IDH-mutant 1p19q codeleted tumors more than 1p19q non-codeleted tumors (**Table 2**).

In the Cox regression model for PFS, the EORTC (HR 0.58, 95% CI 0.3–1.15) and RTOG (HR 0.73, 95% CI 0.32–1.66) risk scores did not significantly impact PFS (**Table 3**).

**Table 3:** Cox regression multivariate analyses of PFS.

Cox regression model of progression-free survival	p-Value	Hazard ratio (95% CI)	BIC	AIC
EORTC overall	0.10	Low *vs*. high 0.58 (0.3–1.15)	229	227
EORTC 1p19q codeleted	0.64	Low *vs*. high 0.73 (0.2–2.7)		
EORTC 1p19q non-codeleted	0.23	Low *vs*. high 0.59 (0.26–1.38)		
RTOG overall	0.74	Low *vs*. High 0.73 (0.32–1.66)	300	398
RTOG 1p19q codeleted	NA	NA		
RTOG 1p19q non-codeleted	0.5	Low *vs*. High 0.7 (0.24–2.04)		
Model 1 - Midline crossing - Post-surgical area (log2)	0.06	Midline crossing Yes *vs*. No 6.36 (0.71–56.9) Post-surgical area (log2) Continuous 1.12 (1.01–1.24)	171	168
Model 1 1p19q codeleted	NA	NA		
Model 1 1p19q non-codeleted	0.7	Midline crossing Yes *vs*. No 2.45 (0.26–22.9) Post-surgical area (log2) Continuous 1.04 (0.91–1.17)		
Model 2 - Midline crossing - Post-surgical area (log2) -Age	0.06	Midline crossing Yes *vs*. No 5.17 (0.57–47.2) Post-surgical area (log2) Continuous 1.12 (1.02–1.24) Age continuous 0.97 (0.93–1.02)	173	168
Model 2 1p19q codeleted	NA	NA		
Model 2 1p19q non-codeleted	0.5	Midline crossing Yes *vs*. No 1.98 (0.21–18.96) Post-surgical area (log2) Continuous 1.04 (0.91–1.17) Age continuous 0.96 (0.9–1.03)		
Model 3 - Midline crossing - Post-surgical area (log2) - Histology	0.1	Midline crossing Yes *vs*. No 5.88 (0.62–55.37) Post-surgical area (log2) Continuous 1.12 (1.01–1.24) Histology (Oligo *vs*. Astro) 0.88 (0.4–1.94)	174	170
Model 4 - Midline crossing - Post-surgical area (log2) - Pre-surgical area (log2)	0.1	Midline crossing Yes *vs*. No 3.73 (0.4–34.9) Post-surgical area (log2) Continuous 1.08 (0.97–1.21) Pre-surgical area (log2) Continuous 1.4 (0.91–2.16)	160	156
Model 4 1p19q codeleted	NA	NA		
Model 4 1p19q non-codeleted		Midline crossing Yes *vs*. No 1.99 (0.20–19.8) Post-surgical area (log2) Continuous 1.05 (0.90–1.23) Pre-surgical area (log2) Continuous 1.3 (0.59–2.78)		

PFS, progression-free survival; AIC, Akaike information criterion; BIC, Bayesian information criterion; EORTC, European Organisation for Research and Treatment of Cancer; RTOG, Radiation Therapy Oncology Group.

When we consider midline crossing and post-surgical area (log2 converted and reported as a continuous variable) in a Cox regression model, post-surgical area confirmed its prognostic role (HR 1.12, 95% CI 1.01–1.24) on PFS, while midline crossing did not (HR 6.36, 95% CI 0.71–56.9; model 1, **Table 3**). Even when corrected with age and histology, residual area (log2 converted and reported as a continuous variable) was the only variable associated with PFS (HR 1.12, 95% CI 1.02–1.24 in model 2; HR 1.12, 95% CI 1.01–1.24 in model 3; reported in **Table 3**). Finally, in a model reporting both pre- and post-surgical areas (log2 converted and reported as a continuous variable) and midline crossing, none of these variables were confirmed to directly impact PFS (model 4, **Table 3**).

We also performed two additional regression models employing age at diagnosis, pre-/post-surgical area log2 converted (or pre-/post-surgical longer diameter), and histology (**Supplementary Table 1**).

### 3.3 Overall survival

At the time of analysis, 39 (63.94%) of 61 patients were alive.

The treatment delivered at the time of progression was surgery alone (n = 13), surgery followed by chemotherapy (n = 4), sequential radio-chemotherapy (n = 19), and radiotherapy (n = 2). Five patients received chemotherapy alone, six patients received radiotherapy alone, and six patients received sequential radio-chemotherapy. The years of diagnosis/study enrolment for each patient in the cohort is reported in the **Supplementary Table 2**).

In univariate analyses, the presence of midline crossing (HR 7.9, 95% CI 1.5–41.55, p = 0.04), astrocytoma histology (HR 2.5, 95% CI 1.02–6.25, p = 0.04), and high-risk EORTC score (HR 4.35, 95% 1.47–12.5, p = 0.04) significantly correlated with shorter survival (**Table 4** and **Supplementary Figure 1**). The EORTC score confirmed its prognostic value, showing a lower BIC/AIC score compared to the RTOG criteria.

**Table 4:** Univariate analysis of overall survival.

Variables	Overall HR (95% CI) p-Value	1p19q Codeleted HR (95% CI) p-Value	1p19q non-codeleted HR (95% CI) p-Value
Gender	2.0 (0.86–4.72) p-Value = 0.1	1.2 (0.83–6.74) p-Value = 0.83	2.15 (0.68–6.8) p-Value = 0.19
Midline crossing	7.9 (1.5–41.55) p-Value = 0.04	NA	5.59 (1.2–31.28) p-Value = 0.05
Age categorical (≤40, >40)	1.68 (0.72–3.89) p-Value = 0.2	0.52 (0.09–2.71) p-Value = 0.43	0.3 (0.09–0.88) p-Value = 0.02
Histology (non-codeleted *vs*. codeleted)	2.5 (1.02–6.25) p-Value = 0.04	NA	NA
RTOG	0.48 (0.11–2.075) p-Value = 0.33	NA	0.81 (0.18–3.65) p-Value = 0.78
EORTC (High *vs*. low)	4.35 (1.47–12.5) p-Value = 0.004	1.66 (0.14–18.71) p-Value = 0.682	0.22 (0.06–0.86) p-Value = 0.03
IDH mutation (non-canonical *vs*. canonical)	0.24 (0.03–1.85) p-Value = 0.09	NA	0.57 (0.07–4.4)
MGMT (unmethylated *vs*. methylated)	1.7 (0.54–5.34) p-Value = 0.36	NA	1.09 (0.33–3.61) p-Value = 0.89
Residual tumor after primary surgery (yes *vs*. no)	1.32 (0.48–3.6) p-Value = 0.6	1.93 (0.21–17.43) p-Value = 0.56	1.06 (0.33–3.42) p-Value = 0.9
Age continuous*	1.09 (0.97–1.04) p-Value = 0.6	1.03 (0.961.11) P = 0.38	1.022 (0.98–1.1) p-Value = 0.32
Pre-surgical perpendicular diameter product log2*	1.62 (0.89–2.93) p-Value = 0.1	0.84 (0.36–1.95) p-Value = 0.85	2.5 (1.23–5.07) p-Value = 0.009
Post-surgical perpendicular diameter product log2*	1.03 (0.90–1.17) p-Value = 0.7	1.05 (0.83–1.32) p-Value = 0.69	1.02 (0.86–1.2) p-Value = 0.82
Re-surgery (yes *vs*. no)	1.28 (0.48–3.38) p-Value = 0.6	0.58 (0.11–2.91) p-Value = 0.51	0.59 (0.16–2.24) p-Value = 0.44
Radiation (yes *vs*. no)	1.1 (0.43–2.79) p-Value = 0.9	0.79 (0.16–3.96) p-Value = 0.78	0.89 (0.27–2.94) p-Value = 0.85
Chemotherapy (yes *vs*. no)	2.16 (0.71–6.58) p-Value = 0.2	2.0 (0.36–11.1) p-Value = 0.43	1.58 (0.34–7.52) p-Value = 0.54
Radiotherapy and adjuvant chemotherapy (yes *vs*. no)	1.06 (0.42–2.69) p-Value = 0.9	0.39 (0.04–3.42) p-Value = 0.39	1.02 (0.31–3.41) p-Value = 0.97
Surgery + adjuvant treatment (radiation and chemotherapy) (yes *vs*. no)	1.3 (0.49–3.41) p-Value = 0.6	0.39 (0.04–3.42) p-Value = 0.39	1.02 (0.31–3.41) p-Value = 0.97

HR, hazard ratio; RTOG, Radiation Therapy Oncology Group; EORTC, European Organisation for Research and Treatment of Cancer; IDH, isocitrate dehydrogenase; MGMT, methylated–DNA–protein–cysteine methyltransferase.

*Continuous variables.

When repeated for both IDH-mutant 1p19q codeleted and IDH-mutant 1p19q non-codeleted tumors, we observed that age, pre-surgical perpendicular diameter product, and EORTC risk score impacted the survival of IDH-mutant 1p19q non-codeleted tumors, while these same variables did not affect the survival of IDH-mutant 1p19q codeleted tumors (**Table 4**).

In a multivariate Cox regression model, we considered initial tumor area (with logarithmic conversion) and histology as variables of interest. In the composed model, both histo-molecular diagnosis (oligodendrogliomas IDH-mutant 1p19q codeleted *vs*. IDH-mutant astrocytoma, HR 0.28, 95% CI 0.10–0.8, p = 0.02) and initial tumor area assessed as continuous variables after logarithmic conversion (HR 1.82, 95% CI 1.01–3.3, p = 0.05) independently affected patient survival (p = 0.01). On the contrary, a composed model including histo-molecular diagnosis and post-surgical diameter product did not demonstrate a statistically significant impact on OS. An additional OS model considering histology, treatment received, second surgery, and pre-/post-surgical area (log2 converted) or pre-/post-surgical longer diameter is reported in **Table 5**.

**Table 5:** Cox regression models for overall survival.

Models (HR, 95% CI, p-value)	HR (95% CI)	p-Value
Model 1		
Re-surgery (yes *vs*. no) Histology (codeletion *vs*. no codeletion)	0.55 (0.2–1.54)	0.26
	0.33 (0.12–0.92)	0.03
Model 2		
Radiation (yes *vs*. no) MGMT (unmethylated *vs*. methylated)	1.4 (0.52–3.74)	0.28
	1.9 (0.58–6.33)	0.5
Model 3		
Radiation (yes *vs*. no) Histology (codeletion *vs*. no codeletion)	1.11 (0.42–3.0)	0.83
	0.44 (0.16–1.2)	0.09
Model 4		
Chemotherapy (yes *vs*. no) MGMT (unmethylated *vs*. methylated)	1.94 (0.68–5.56)	0.22
	2.1 (0.62–7.0)	0.23
Model 5		
Chemotherapy (yes *vs*. no) Histology (codeletion *vs*. no codeletion)	1.36 (0.48–3.9)	0.57
	0.46 (0.17–1.24)	0.12
Model 6		
Radio-chemotherapy (yes *vs*. no) MGMT (unmethylated *vs*. methylated)	1.95 (0.75–5.04)	0.16
	1.95 (0.59–6.43)	0.27
Model 7		
Radio-chemotherapy (yes *vs*. no) Histology (codeletion *vs*. no codeletion)	1.41 (0.54–3.7)	0.48
	0.47 (0.17–1.2)	0.14

HR, hazard ratio; MGMT, methylated–DNA–protein–cysteine methyltransferase.

## 4 Discussion

Prognostic stratification is crucial for the appropriate clinical management of patients with adult-type diffuse glioma IDH mutant. This study investigated the prognostic hallmarks of adult-type diffuse glioma IDH mutant grade 2 through a correlative analysis of clinical, radiological, histological, and molecular features. The study population was selected according to the following key characteristics: 1) patients underwent an active surveillance approach after initial surgery and 2) included subtypes that met the diagnostic histological and molecular criteria of the WHO classification published in 2021. Improving prognostic tools for low-grade gliomas is a critical need in neuro-oncology to allow the selection of patients more likely to benefit from targeted therapy. Furthermore, the recently updated central nervous system (CNS) tumor classification requires the re-evaluation of clinical prognostic scores for grade 2 IDH-mutant glioma (EORTC and RTOG scores), established in the pre-molecular era (8, 10).

In a large series of WHO 2021-defined adult-type diffuse glioma IDH mutant, Harvey-Jumper et al. identified three risk groups for PFS: all IDH-mutant 1p19q codeleted oligodendrogliomas were associated with lower risk, regardless of the extent of surgery (34). Recently, long-term results from the observation arm of the RTOG 9802 (patients with age <40 years and complete resection) confirmed different survival outcomes for IDH-mutant astrocytomas compared to IDH-mutant 1p19q oligodendrogliomas (median PFS 2.8 years *vs*. 8.3 years, p < 0.001) in a cohort of untreated patients. Compared to this cohort, our study population had less favorable postoperative characteristics, explaining the different median PFS outcomes (11, 35, 36).

The prognostic impact of residual tumor after resection has been addressed by several retrospective studies, and maximal safe resection remains the cornerstone of current practice in adult-type diffuse glioma IDH mutant. In our cohort, the choice of partial resections for most patients was due to tumor location and extension in eloquent areas to avoid permanent functional damage after surgery. In our study, residual tumor after resection had a significant impact on PFS and included the major extent of residual tumor area as a continuous variable in univariate analyses.

While some literature data report a benefit of larger resections in all IDH-mutant gliomas, other retrospective series have reported a benefit only in the IDH-mutant astrocytoma subgroup (21, 37–39). Unequivocal conclusions have long been challenged by the marked heterogeneity in study populations, particularly in patient cohort characteristics (e.g., histological grading, diagnostic assessment, and treatment methodologies). Heterogeneity in study methods (survival endpoints) and in methods to assess tumor extent (tumor area and tumor volume) should also be considered. Following the WHO reclassification of gliomas in 2016, several retrospective studies have attempted to confirm a rationale for a greater extent of resection across molecularly defined subtypes. In a retrospective series of gliomas diagnosed according to the 2016 WHO classification by Wijnenga et al., the authors found that postoperative volume was associated with OS, with a strong detrimental effect of even small tumor remnants only in IDH-mutant astrocytomas, but not in IDH-mutant 1p19q codeleted oligodendrogliomas (22). In another recent large retrospective study, the extent of resection did not affect OS outcomes among IDH-mutant 1p19q codeleted oligodendroglioma patients with non-enhancing disease (18). The hypothesis was that residual tumor would have less impact on outcome in oligodendroglioma due to greater sensitivity to radiotherapy and chemotherapy.

Overall, studies including adult-type diffuse glioma IDH mutants after the 2021 WHO reclassification have suggested that multiple factors interact to shape disease risk. Determining the weight of each of these factors and integrating them into the decision algorithm is the current challenge.

In our series, residual tumor affected PFS but did not appear to affect OS, although we observed a limited number of events for OS analyses. In a recent study, Van der Vaart et al. reported integrated molecular diagnosis of IDH-mutant astrocytoma versus IDH-mutant 1p19q codeleted oligodendrogliomas, with pre- and postoperative tumor volume as independent prognostic factors for survival. Consistent with previous series, the impact of postoperative tumor volume on survival was greater in the IDH-mutant astrocytoma subgroup than in the IDH-mutant 1p19q codeleted oligodendroglioma subgroup. In contrast to our study, all patients with adult-type diffuse glioma IDH mutant were included, regardless of grade. However, pre- and postoperative tumor dimensions had a greater impact on survival than tumor grade or the presence of enhancement (21).

In our study, the immaturity of the OS data made it difficult to draw further conclusions about survival determinants. However, in univariate OS analyses, the EORTC “high-risk” category significantly correlated with shorter survival. We speculate that this may support the greater impact of baseline tumor dimensions.

According to recent literature, it would be possible that tumor dimension may reflect a higher likelihood of developing a more aggressive neoplastic clone through increasing acquisition of genetic alterations (40).

Limiting these observations were the relatively small number of OS events and the inability to obtain a volume estimate of the tumoral mass. In addition, multivariate models employing more than three variables did not allow us to confirm the proportional axiom.

Another limitation of the study was the lack of data regarding the patients' performance status prior to surgery. The patients selected were assessed at our center after primary surgery and were able to undergo oncological treatment, presenting an ECOG score of 0 or 1. The decision not to administer adjuvant therapies after surgery was due to delays in post-surgical evaluation or for historical reasons, e.g., differences in therapeutic approaches based on the year of diagnosis.

Consistent with our hypothesis of the greater impact of baseline tumor dimension, the validation of risk factors from the EORTC prognostic index in a cohort of adult-type diffuse glioma IDH-mutant patients showed that superior PFS and OS in the “low-risk” group were primarily due to the influence of histology and baseline tumor size (26). Similarly, in a cohort of untreated, molecularly characterized adult-type diffuse glioma IDH-mutant patients, tumor diameter >6 cm and midline crossing were identified as independent prognostic factors for PFS among the items included in the EORTC score, highlighting the major impact of tumor size on risk assessment (41).

In a cohort of patients reclassified according to the 2021 WHO, preoperative tumor size was an independent predictor of survival in both the IDH-mutant astrocytoma and IDH-mutant 1p19q codeleted oligodendroglioma subgroups (42). Tom et al. investigated risk factors for tumor progression in patients with adult-type diffuse glioma IDH mutant who underwent a watch-and-wait approach after gross tumor resection: increasing age, larger initial tumor size, and IDH-mutant/1p19q non-codeleted cases demonstrated a detrimental impact on PFS (43). In contrast, age had no effect on survival in our series, which may be explained by the large proportion of elderly patients among IDH-mutant 1p19q mutant oligodendroglioma patients in our cohort. Overall, our data are consistent with previous studies confirming a very limited effect of age and, in particular, of the 40-year cut-off on survival (42).

We expect that future studies will clarify whether tumor extent has a biological significance and, eventually, which biological changes occur in tumors with higher disease burden (44, 45).

A prognostic multidimensional approach is likely to be essential to predict the outcome of adult-type diffuse glioma IDH mutant at an individual level in order to select appropriate management. In a recent integrated summary of recommendations according to WHO 2021, Kotecha et al. proposed the identification of three different risk categories (low, intermediate, and high). In the proposed algorithm, the low-risk group was identified as candidates for a “watch and wait” approach. In this context, long-term follow-up data from the RTOG 9802 observational arm, published in 2022, encouraged a better characterization of patients with extremely favorable prognosis, as almost one-third of patients had no disease progression at 15 years (46). Consistent with data from the INDIGO trial, even patients with residual tumor in the absence of contrast enhancement and clinical risk factors (e.g., functional deficits, uncontrolled seizures, and need for steroid therapy) could benefit from vorasidenib (46, 47).

Although our results are similar to previously published data, our study addresses some important issues: integrated diagnosis according to WHO 2021 is essential to define adult-type diffuse glioma IDH-mutant subgroups and to explore key characteristics of each subtype; also, since patients with grade 2 glioma IDH mutant have a median survival of >10 years, our extended follow-up allows reliable data interpretation. Further follow-up and expansion of the dataset will be important for definitive conclusions.

The main limitation of our study is its retrospective nature; however, ethical and practical issues do not allow a prospective study to enroll patients for a watchful waiting approach without consideration of established risk criteria. In our opinion, retrospective series with detailed clinical and molecular data and long-term follow-up are the best available research strategy. Another important limitation is the inability to assess the presence/absence of cyclin-dependent kinases (CDKN2A/B) in most of the patients in the cohort. To mitigate this limitation, an expert neuropathologist confirmed that all pathological specimens had a molecularly confirmed IDH1 or 2 mutation and low-grade histo-pathological features. However, we did not include in our case series any patient with a known homozygous CDKN2A/2B deletion. According to the previous series, the possibility of finding a CDKN2A/B homozygous deletion is less than 10% in IDH-mutant gliomas (45). In addition, the positive trend in terms of PFS and OS of the whole cohort correlates with tumors without CDKN2A/B deletion.

Another limitation of the study is the inability to include growth rate as a variable in our analysis. Growth rate reflects tumor aggressiveness and may correlate with underlying molecular features. Unfortunately, in our study, imaging data and software did not allow for consistent, quantitative measurement of tumor growth. In addition, data on seizure control during the clinical course were not available for most of the patients.

In the future, we expect that the integration of comprehensive clinical profiles and molecular characterization will allow for the accuracy of data interpretation. Moreover, multicenter collaborations would enable the collection of larger and more diverse patient cohorts, thereby increasing the statistical power and generalizability of findings. Such collaborative efforts could also harmonize methodological approaches, reduce biases, and facilitate the validation of biomarkers and prognostic factors.

## 5 Conclusions

Novel prognostic models should be explored to obtain a better estimation of prognosis and disease-free survival in the molecular era. The presence or absence of 1p19q codeletion influences the survival of these patients.

Our study enforces the impact of clinical parameters on estimating prognosis and raises questions about biological correlates for clinically high-risk patients. In our series of adult-type diffuse gliomas IDH mutant under active surveillance after primary surgery, midline crossing, presence of residual tumor, and extent of residual tumor affected progression-free survival.

## Data Availability

The raw data supporting the conclusions of this article will be made available by the authors, without undue reservation.

## Supplementary material

Supplementary Figure 1 Kaplan Maier curves for overall survival according to **(A)** presence absence of midline crossing, **(B)** Histology, and **(C)** EORTC risk subgroup.

Supplementary Table 1 Multivariate analyses for progression free survival.

Supplementary Table 2 Years of diagnosis/study enrolment of patients in study cohort.
