# Supplementary figures and images for: How to predict progression-free survival in patients with grade 2 IDH-mutated diffuse gliomas after surgery: a long-term follow-up analysis
Source: Front Oncol. 2025 Nov 18;15:1673285. doi: 10.3389/fonc.2025.1673285 (PMC12668962; doi:10.3389/fonc.2025.1673285)

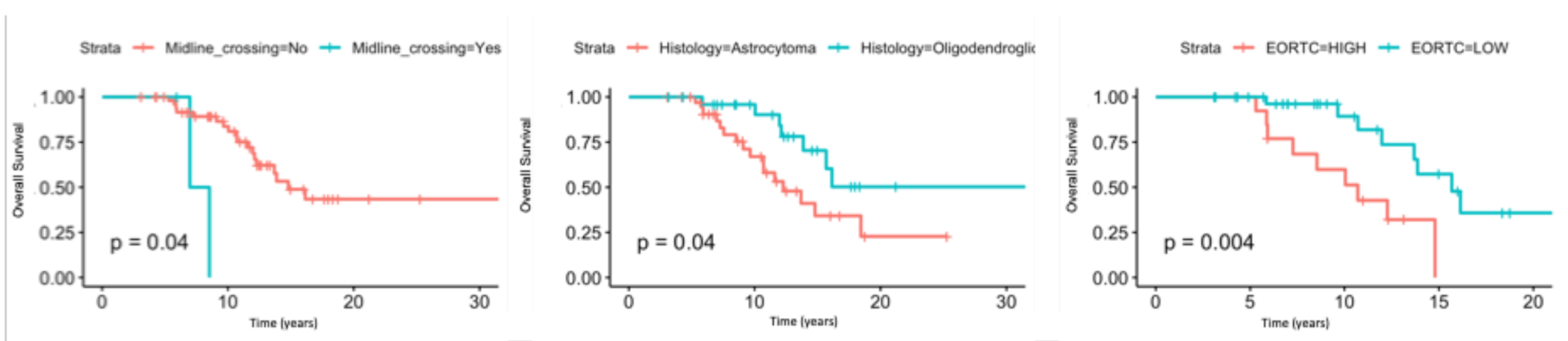

Supplement: Supplementary Figure 1 — Kaplan Maier curves for overall survival according to (A) presence absence of midline crossing, (B) Histology, and (C) EORTC risk subgroup. [file Image1.png]
