# Supplementary material for: How to predict progression-free survival in patients with grade 2 IDH-mutated diffuse gliomas after surgery: a long-term follow-up analysis
Source: Front Oncol. 2025 Nov 18;15:1673285. doi: 10.3389/fonc.2025.1673285 (PMC12668962; doi:10.3389/fonc.2025.1673285)
Supplement: Supplementary Table 1 — Multivariate analyses for progression free survival. [file Table1.docx]

| **Variable** | **HR (95%CI)** | **P value** |
| --- | --- | --- |
| Histology (No codeletion vs codeletion) | 1.2 (0.52-2,81) | 0.66 |
| Age diagnoses | 0.95 (0.91-1.01) | 0.06 |
| Pre-tumoral area log | 1.49 (0.96-2.3) | 0.07 |
| Post tumoral area log | 1.1 (0.97-1.2) | 0.15 |

| **Variable** | **HR (95%CI)** | **P value** |
| --- | --- | --- |
| Histology (No codeletion vs codeletion) | 1.4 (0.61-3,33) | 0.41 |
| Age diagnoses | 0.96 (0.91-1.01) | 0.13 |
| Pre-tumoral diam max | 0.97 (0.94-1.01) | 0.13 |
| Post tumoral diam max | 1.04 (1.01-1.1) | 0.03 |
