# Supplementary material for: How to predict progression-free survival in patients with grade 2 IDH-mutated diffuse gliomas after surgery: a long-term follow-up analysis
Source: Front Oncol. 2025 Nov 18;15:1673285. doi: 10.3389/fonc.2025.1673285 (PMC12668962; doi:10.3389/fonc.2025.1673285)
Supplement: Supplementary Table 2 — Years of diagnosis/study enrolment of patients in study cohort. [file Table2.docx]

| **TREATMENT** | **HR** | **P-value** |
| --- | --- | --- |
| Re-surgery | 1.28  (0.48-3.38) | 0.6 |
| Radiation | 1.1  (0.43-2.79) | 0.9 |
| Chemotherapy | 2.16  (0.71-6.58) | 0.2 |
| Radiotherapy and adjuvant Chemotherapy | 1.06  (0.42-2.69) | 0.9 |
| Surgery + Adjuvant Treatment (radiation and chemotherapy) | 1.3  (0.49-3.41) | 0.6 |
